# The Role of Ketamine in the Treatment of Bipolar Depression: A Scoping Review

**DOI:** 10.3390/brainsci13060909

**Published:** 2023-06-04

**Authors:** Muhammad Youshay Jawad, Saleha Qasim, Menglu Ni, Ziji Guo, Joshua D. Di Vincenzo, Giacomo d’Andrea, Aniqa Tabassum, Andrea Mckenzie, Sebastian Badulescu, Iria Grande, Roger S. McIntyre

**Affiliations:** 1 Mood Disorders Psychopharmacology Unit, University Health Network, Toronto, ON M5T 2S8, Canada; youshayjwd@gmail.com (M.Y.J.); salehaqasim96@gmail.com (S.Q.); joshua.divincenzo@uhnresearch.ca (J.D.D.V.); aniqa.tabassum@uhn.ca (A.T.); andrea.mckenzie@uhn.ca (A.M.); sebastian.badulescu@mail.utoronto.ca (S.B.); 2 Institute for Mental Health Policy Research, Centre for Addictions and Mental Health, Toronto, ON M6J 1H4, Canada; 3 Department of Pharmacology and Toxicology, University of Toronto, Toronto, ON M5S 1A8, Canada; menglu.ni@mail.utoronto.ca (M.N.); ziji.guo@mail.utoronto.ca (Z.G.); 4 Department of Psychology, University of Toronto, Toronto, ON M5S 3G3, Canada; 5 Brain and Cognition Discovery Foundation, Toronto, ON M5S 1M2, Canada; 6 Department of Neuroscience, Imaging and Clinical Sciences, University “G. d’Annunzio”, 66100 Chieti, Italy; giacomo.dandrea1993@gmail.com; 7 Bipolar and Depressive Disorders Unit, Hospital Clinic de Barcelona, C. Villarroel, 170, 08036 Barcelona, Spain; igrande@clinic.cat; 8 Departament de Medicina, Facultat de Medicina i Ciències de la Salut, Universitat de Barcelona (UB), C. Casanova, 143, 08036 Barcelona, Spain; 9 Institut d’Investigacions Biomèdiques August Pi i Sunyer (IDIBAPS), C. Villarroel, 170, 08036 Barcelona, Spain; 10 Institute of Neurosciences (UBNeuro), P. de la Vall d’Hebron, 171, 08035 Barcelona, Spain; 11 Centro de Investigación Biomédica en Red de Salud Mental (CIBERSAM), Instituto de Salud Carlos III, 28029 Madrid, Spain; 12 Department of Psychiatry, University of Toronto, Toronto, ON M5T 1R8, Canada

**Keywords:** ketamine, bipolar depression, mood disorders, psychedelics, bipolar disorder

## Abstract

Bipolar depression remains a clinical challenge with a quarter of patients failing to respond to initial conventional treatments. Although ketamine has been extensively studied in unipolar depression, its role in bipolar disorder remains inconclusive. The aim of our scoping review was to comprehensively synthesize the current clinical literature around ketamine use in bipolar depression. A total of 10 clinical studies (5 randomized controlled trials and 5 open label studies) were selected. The preliminary evidence, albeit weak, suggests that ketamine is a promising treatment and calls for further interest from the research community. Overall, ketamine treatment appeared to be tolerable with minimal risk for manic/hypomanic switching and showed some effectiveness across parameters of depression and suicidality. Moreover, ketamine is a potential treatment agent in patients with treatment-resistant bipolar depression with promising data extracted from extant controlled trials and real-world effectiveness studies. Future studies are needed to identify ketamine’s role in acute and maintenance treatment phases of bipolar depression. Moreover, future researchers should study the recurrence prevention and anti-suicidal effects of ketamine in the treatment of bipolar depression.

## 1. Introduction

Bipolar disorder (BD) is a serious and debilitating psychiatric disorder affecting multiple domains of mood, emotions, and cognition [1]. Overall, BD has a global prevalence of >1% and constitutes a major healthcare burden [2,3]. Patients with BD often have two distinct mood episodes, i.e., mania/hypomania and depression [4]. Using the criteria for mood episodes and their duration, severity, and occurrence, BD is broadly classified into BD-I, BD-II, and cyclothymia in the Diagnostic and Statistical Manual of Mental Disorders (DSM) 5th edition [1].

BD-I is diagnosed following a manic episode, in which the individual may feel a significant increase in energy or irritability in mood for a week or longer; BD-II involves depressive and euthymic phases, along with hypomanic phases, which are characterized by less severe manic symptoms that need only last four days in a row rather than a week; and cyclothymic disorder, which is a milder form of bipolar disorder involving many “mood swings” with hypomania and depressive symptoms that occur rather frequently [4,5]. In addition to DSM criteria, individuals with BD often present on a spectrum of multiple clinical symptoms (e.g., mood, cognition, emotions, etc.) and patient-reported outcomes (e.g., sleep, fatigue, occupational deficits, etc.) [4,6]. Moreover, suicidality remains one of the most serious consequences of BD. It is estimated that suicide rates for patients with BD are approximately 20 times higher than the general population, with a quarter of them resulting in serious harm [7,8,9]. 

Though mania/hypomania are the defining features of BD, patients typically spend relatively more time experiencing depressive symptoms [4,10]. Despite the significant burden of bipolar depression, there are only a handful of Food and Drug Administration (FDA)-approved treatments for this specific condition (i.e., olanzapine and fluoxetine combination, quetiapine, lurasidone, cariprazine, and lumateperone) [11]. The mechanisms of these pharmacotherapies are diverse and multifaceted, as many act on multiple receptors at a time and in unique ways, which reflects our incomplete understanding of the pathophysiology of BD. For example, quetiapine is mainly an inhibitor of 5-HT_2a_ and D_2_ receptors, but also acts on D_1_ and H_1_ histamine receptors, among others, contributing to its adverse effects (e.g., drowsiness, weight gain) [12]; olanzapine is an inverse agonist of 5HT_2a_ receptors, antagonist of D_1_, D_2_, D_3_, and D_4_ dopamine receptors, H_1_ receptors, and also binds to alpha-1 adrenergic and some postsynaptic muscarinic receptors [13], with similar adverse effects to quetiapine; and lumateperone is a newer agent which acts as a 5-HT_2a_ antagonist, D_2_ presynaptic partial agonist and postsynaptic antagonist, serotonin reuptake inhibitor, and glutamate modulator with fewer off-target effects and a more favorable side-effect profile compared to previous antipsychotics [14,15]. Moreover, lithium, which is the oldest pharmacotherapy for managing BD, has a poorly understood mechanism of action, yet is still a first-line treatment when it comes to suicidality and rapid mood cycling [16]. It has been estimated that 25% of patients are resistant to current treatments for bipolar depression, posing huge economic, clinical, and personal burdens [17]. In view of this, there is an urgent need to develop treatment modalities that can help in not only remitting depressive symptoms among those who fail to respond to initial treatments but can also help in tackling any residual symptoms, often pertaining to cognition [18].

The pathophysiological mechanisms underlying bipolar disorder involve abnormalities in various specific brain regions. For example, GABAergic inhibitory circuits in the cortex are known to be dysfunctional in patients with BD, and these deficits can be reversed with mood stabilizers such as lithium, valproate, and atypical antipsychotics such as olanzapine [19]. Indeed, it could be posited that GABAergic receptor-mediated inhibitory deficits in BD could increase the proclivity for disinhibited behaviors (i.e., risk taking, hedonism, and sleeplessness). Such a relationship has been previously reported in healthy subjects with greater anxiety traits [20] and in patients with schizophrenia experiencing greater psychotic symptoms [21]. The hippocampus is another key brain region implicated in BD pathophysiology, and is a nexus for learning, memory, and cognition. Preclinical studies using mouse models for psychiatric disorders have found that hippocampal dentate gyrus cells were arrested at a “hyperexcitable” stage with similar molecular and physiological properties to those of the immature neurons, conferring working memory deficits and hyper-locomotor activity, which has been posited as an endophenotype of BD [22,23,24,25]. Thus, with both above examples converging on increased glutamatergic activity by aberrant inhibitory mechanisms, modulating intermediaries of glutamatergic signaling, such as N-methyl-D-aspartic acid (NMDA) and α-amino-3-hydroxy-5-methyl-4-isoxazolepropionic acid (AMPA) receptors could be promising targets for treating BD.

In the last decade, research on N-methyl-D-aspartic acid (NMDA) antagonists (i.e., ketamine and esketamine) has shown promising results in managing treatment-resistant unipolar depression [26,27]. Though ketamine infusion is not FDA-approved for depression treatment, its S-enantiomer (i.e., esketamine) has been FDA-approved for managing treatment-resistant depression [26]. Key benefits of ketamine and esketamine include the rapid-acting antidepressant effects (i.e., within hours of an intravenous infusion) [28], and the absence of metabolic side effects compared to other pharmacotherapies for bipolar depression such as olanzapine/fluoxetine combination therapy, which causes weight gain and can take 4–8 weeks before it is effective [1]. Moreover, IV ketamine produces rapid acting (within 2-, 4-, and 24-h post-dose) anti-suicidal effects that can last for up to a week, which offers the potential of a promising intervention for treating severe acute suicidal ideation [29], especially important for BD given the aforementioned high burden of suicidality. However, evidence supporting the use of ketamine/esketamine in bipolar depression remains inconclusive [11]. There is some preclinical evidence that glutaminergic agents can act not only as antidepressants, but also as mood stabilizing agents in patients with BD [30]. However, clinical studies that could confirm and expand this preclinical notion remain to be conducted.

As such, our scoping review is focused on synthesizing the available literature on the use of ketamine/esketamine in bipolar depression, with a focus on its safety and efficacy/effectiveness across multiple domains (i.e., depressive symptoms, suicidality, cognition, and anxiety). Furthermore, we set forth to highlight research and clinical gaps that could help future researchers to study ketamine treatment in BD better.

## 2. Methods

This review was conducted in accordance with the guidelines of Preferred Reporting Items for Systematic reviews and Meta-Analyses (PRISMA) for scoping reviews [31]. The protocol was registered as a priority on the open science framework (OSF; DOI 10.17605/OSF.IO/CS6TJ).

### 2.1. Eligibility Criteria

The present study aimed to synthesize the clinical literature on the use of ketamine or esketamine in the treatment of bipolar depression. As such, we included all studies that delineated the efficacy/effectiveness or safety of ketamine use in patients with BD. Since bipolar and unipolar depression differ in their patient characteristics, clinical presentation, treatment strategies, and prognosis, we excluded studies that did not distinguish between these two phenotypes [1,32,33]. Hence, the studies that are composed only of patients with BD were included herein (both BD-I and BD-II). Moreover, studies were required to report at least one outcome directly related to clinical practice (i.e., change in depression, anxiety, or suicidality measurements or adverse/tolerability effects). The efficacy/effectiveness measures needed to be measured on standardized clinical scales (e.g., Montgomery–Åsberg Depression Rating Scale (MADRS), Scale for Suicide Ideation (SSI), etc.) [17,32]. Efficacy was defined as the group level change in the measured domain (e.g., depression, suicidality, anxiety, etc.) under ideal and controlled conditions. On the other hand, effectiveness was defined as change in measured psychological domains from group baseline in a real-world or open label scenarios.

Additionally, included studies were required to be original research reporting quantitative results; qualitative, abstracts, case reports/series, and secondary sources were excluded. 

In accordance with the PRISMA statement [31], the foregoing criteria were formalized in the following PIECOS outline:

Population: Adults aged 18 years and older.

Intervention: Intravenous (IV) racemic ketamine, intranasal (IN) esketamine, or IV arketamine.

Exposures: Clinical diagnosis of BD according to DSM-IV or 5 editions.

Comparison group(s): placebo or none.

Outcome(s): Studies must assess at least one clinically relevant factor (i.e., efficacy, effectiveness, tolerability, or adverse effects) across one or more domains of bipolar depression (i.e., depressive symptoms, cognition, anxiety, and suicidality). 

Studies: Quantitative and original studies of direct clinical relevance such as: randomized controlled trials (RCTs), open-label studies, retrospective chart reviews, or observational clinical studies. 

### 2.2. Search Strategy and Study Inclusion

A comprehensive search of online databases (i.e., MEDLINE(R), Embase Classic + Embase, APA PsycINFO, Ovid Healthstar, Journal@Ovid Full Text, Cochrane, Google Scholar, and CINAHL) was completed from inception to 28 April 2023, using the following search string: ((bipolar disorder) OR (bipolar depression)) AND (((arketamine) OR (esketamine)) OR (ketamine)). Database search results were imported into the Covidence platform (https://www.covidence.org/) for deduplication, screening, and risk of bias assessment. Two reviewers (M.Y.J., S.Q.) independently screened the imported titles and abstracts, then assessed the remaining full texts for eligibility. Conflicts in judgment were resolved by discussion with a third reviewer (R.S.M.). 

### 2.3. Data Extraction and Analysis

The following data points were extracted in a pilot-test Excel table wherever possible: lead author, study design, study participants, intervention (and control, if any), primary objectives, main findings, and limitations. Efficacy/effectiveness was defined as difference in measured domain (e.g., depression, suicidality, etc.) from baseline measured on standardized clinical scales (such as Hamilton Depression Rating Scale, Scale for Suicide Ideation, etc.). Safety, tolerability data and switching to mania/hypomania, and discontinuation rates were operationalized as adverse effects with an associative statistic (e.g., odds ratio, prevalence, etc.). The extracted data points were synthesized qualitatively across the lines of efficacy/effectiveness in ameliorating depressive or suicidality symptoms, overall tolerability, and risk for mania/hypomania.

### 2.4. Risk of Bias Assessment

Assessments of methodological quality were independently conducted by two reviewers (M.Y.J. and S.Q.) using Cochrane’s risk of bias tools [34]. Since we included both blinded RCTs and unblinded follow-up/observational studies or open label trials, the standard Risk of Bias (RoB) tool for randomized-controlled trials and the tool for Risk of Bias in Non-randomized Studies of Interventions (ROBINS-I) were applied, respectively [35,36].

## 3. Results

### 3.1. Search Results 

**Figure 1:**
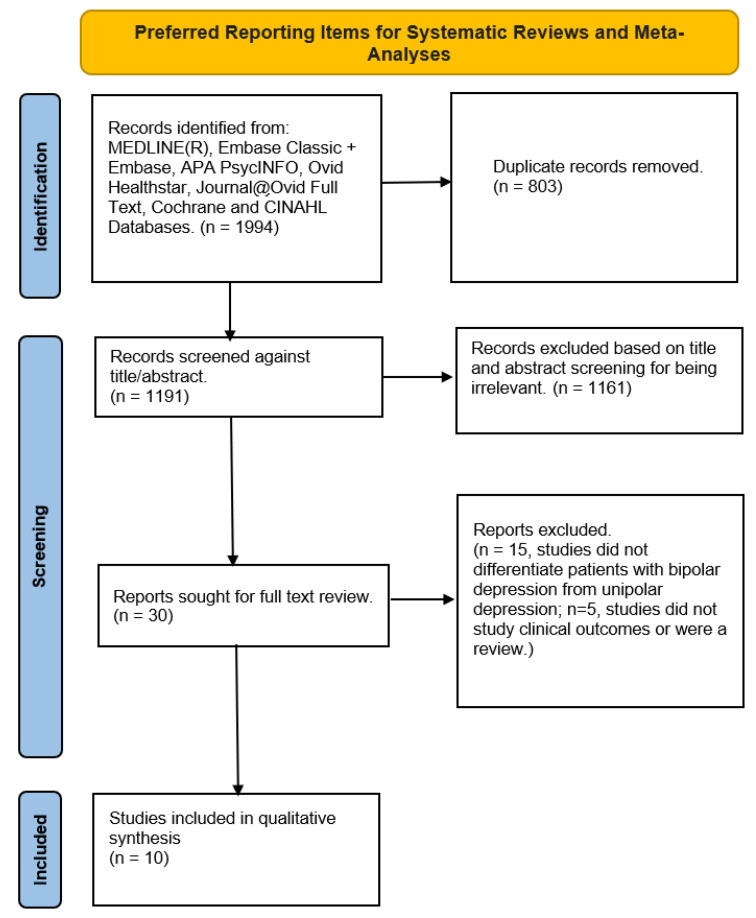
Preferred Reporting Items for Systematic Reviews and Meta-Analyses (PRISMA) extension for scoping review flowchart for included studies.

A total of 1994 studies were identified in our initial search. After the removal of duplicates, 1191 studies were left to screen against title and abstract. Following title and abstract screening, 30 studies were eligible for full text analysis and 10 studies were included in the present review. Details relevant to study selection are outlined in Figure 1.

### 3.2. Study Characteristics and Quality Appraisal

As a whole, our review identified 5 RCTs and 5 open label trials investigating the role of IV ketamine in BD. We did not find any study using IN esketamine or IV arketamine in managing depressive symptoms across bipolar depression. The most commonly used dosage was 0.5 mg/kg. Almost all participants in open label studies concurrently used various mood stabilizing agents (e.g., lamotrigine, valproate, lithium, etc.) or antipsychotics (e.g., olanzapine, quetiapine, etc.). Two out of five RCTs (Grunebaum et al. and Abbar et al.) allowed participants to continue with their usual medications while the remaining three RCTs allowed the participants only to take lithium and valproate. A few detailed characteristics of studies are delineated in Table 1, including lead author, study type, total participants, dosage and mode of intervention, dosage and mode of control (for RCTs), concurrent medications, psychometric tools being used, primary objectives, findings, reports of mania/hypomania, and limitations. 

Overall, the quality appraisal of included studies showed concerns across multiple domains of robustness. The component studies had a small number of participants that could have led to limited statistical power to identify true efficacy/effectiveness of IV ketamine use in BD patients. Moreover, studies reported on acute effects of IV ketamine use (from 24 h to one week) and lacked the ability to provide information for long-term clinical management. The reported follow-up period in most studies was two to three weeks, limiting the validity of results in long-term usage. The ROBINS-I assessment showed moderate concerns for confounding since patients were on multiple psychopharmacological treatments with no control to measure the effectiveness robustly. The participants included in many studies were hospitalized or had treatment-resistant disease which limits the generalizability of results. Most studies either did not blind the individuals conducting the outcome assessment (standardized self-reported depression scoring and cognitive tests) or lacked information about blinding, leading to a moderate risk of bias in outcome measurement. Moreover, the majority of open label trials had concerns due to participants dropping out. The lack of control group made it difficult to rule out placebo effect completely. 

**Figure 2:**
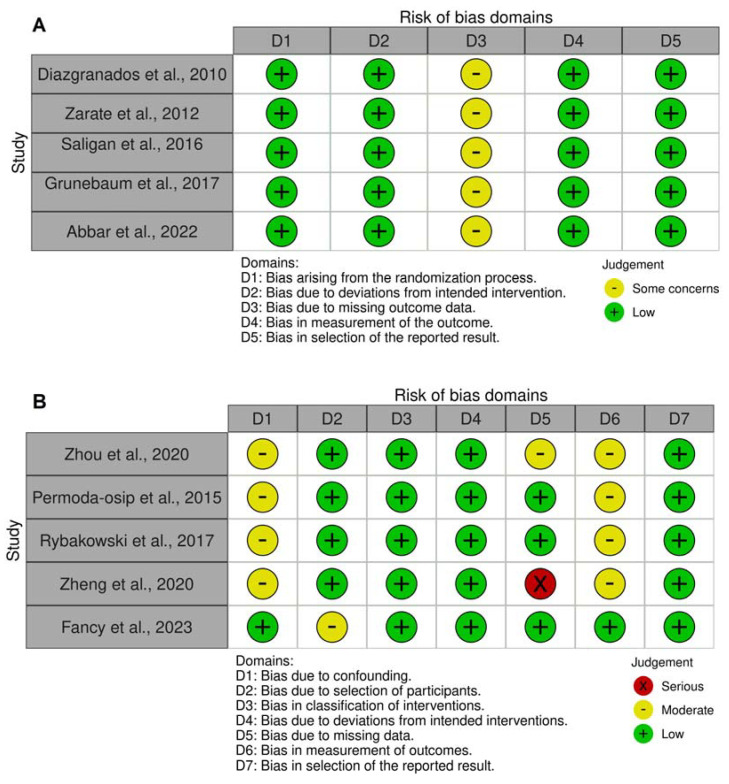
Risk of bias assessment results for the included literature at the domain level, wherein (**A**) shows results for randomized controlled studies as per Cochrane’s Risk of Bias tool (RoB), and (**B**) shows results for open label studies as per Cochrane’s Risk of Bias in Non-randomized Studies-of Interventions (ROBINS-I) tool. Icons were generated using the open-source RobVis ShinyApp: https://www.riskofbias.info/welcome/robvis-visualization-tool.

On the other hand, all RCTs showed some concerns for missing data as assessed through the ROB tool for RCTs. Moreover, none of the RCTs reported on the post-trial statistics for the success of blinding that could have led to higher placebo rates in interventional groups. The detailed domain level results for the quality appraisal are presented in Figure 2, and further limitations of each constituent study are delineated in Table 1. It was noted that even though most studies showed a significant decrease in depression scores following ketamine infusion, the effect size varied. Hence, the response rates and remission rates are discussed separately in the review and should be accounted for while deriving conclusions.

### 3.3. Efficacy of Ketamine Use in Bipolar Depression

Our search identified 5 RCTs that delineated the efficacy of IV racemic ketamine in the treatment of acute bipolar depression. The crossover double blinded RCT by Diazgranados et al. randomized 18 participants with BD (concurrent treatment with lithium or valproate) to receive one infusion of ketamine (0.5 mg/kg) or normal saline (as control) [37]. The study showed a statistically significant reduction in depression among ketamine users when compared to placebo (d = 0.52, 95% CI= 0.28–0.76, *p* < 0.05) after 40 min of infusion. The antidepressant effect remained statistically differentiated from placebo subgroups for up to three days post-infusion. The maximum response rates (50% reduction in depressive measurement) were 71% and 6% among ketamine and placebo subgroups up to three days post-treatment, respectively [37]. Another RCT with similar patient characteristics and study design reported an antidepressant effect among 15 participants receiving IV ketamine when compared to placebo (d = 0.89, 95% CI = 0.61–1.16, *p* < 0.05) 40 min post-infusion. The study reported approximately similar response rates compared to previous RCT (i.e., 79% in the ketamine group and 0% in the placebo group) over the period of the trial. Moreover, anti-suicidal effects were observed with the use of ketamine when compared to placebo in the same study (d = 0.98, 95% confidence interval = 0.64–1.33, *p* < 0.05) 40 min post-infusion [38]. However, an RCT by Grunebaum et al. in 16 BD patients did not show any statistically significant anti-suicidal effect of IV ketamine compared to IV midazolam (control) [39]. This was the only RCT that used IV midazolam as control while studying patients with BD and suicidal ideations. Notwithstanding, a recent RCT by Abbar et al. comprising 52 BD patients with suicidality reported a statistically significant resolution of suicidal symptoms (Scale for Suicide Ideation measurement < 4) among ketamine users when compared to control (IV saline) by day 3 (OR = 14.1, 95%CI = 3.0 to 92.2, *p* < 0.001) [40].

Lastly, an RCT by Saligan et al. reported on anti-fatigue properties of IV ketamine among 36 patients with bipolar depression maintained on either lithium or valproate. When controlled for non-fatigue depressive symptoms (such as anhedonia, hopelessness, psychomotor retardation, etc.), the study suggested a statistically significant difference measured on NIH-Brief Fatigue Inventory between patients randomized to ketamine and placebo (IV normal saline) with the highest difference on day two post-treatment (d = 0.58, *p* < 0.05) [41].

In a nutshell, the small number of participants in the included RCTs makes the scientific literature severely limited related to the role of ketamine treatment in acute and/or maintenance treatment of bipolar depression (with or without suicidality), relevant patient characteristics (gender and degree of bipolar depression), and combination treatment with mood stabilizing agents (e.g., lithium, lamotrigine, etc.). Moreover, in all RCTs, patients were tapered off their ongoing psychopharmacological treatments to receive mood stabilizers only. This could have led to fluctuation in their depressive measures and in turn, measurement bias in outcome measures. 

### 3.4. Effectiveness of Ketamine Use in Bipolar Depression

A total of 5 studies were identified that investigated the use of IV ketamine in an open label or retrospective clinical setting. An in-patient interventional study by Rybakowski et al. investigated the role of ketamine among 53 patients with bipolar depression taking >1 mood stabilizing agents (i.e., carbamazepine, lithium, quetiapine, valproate, lamotrigine, quetiapine, aripiprazole, and topiramate). Almost half of the participants showed response to ketamine treatment (i.e., *n* = 27; 51%) with more males than females (i.e., 77% > 43%) after 7 days of treatment [42]. Another in-patient interventional study by Zhou et al. delineated the effects of nine ketamine infusions over the period of 3 weeks among 38 patients with bipolar depression. The findings suggested a significant decrease in depressive symptoms after one week. However, the patients gradually relapsed into depression in the proceeding two weeks with pre-treatment depressive measures after three weeks of the study period [43]. Nevertheless, a recent open label study by Zheng et al. consisting of 6 ketamine infusions across 12 days suggested significant effectiveness of ketamine treatment among patients with bipolar depression even after study completion. Among 16 patients, 73.7% and 63.2% showed response and remission rates after 12 days, respectively [44]. 

A recent real-world study from Canada investigated the use of IV racemic ketamine (0.5–0.75 mg/kg; 4 infusions) over a two-week period among 66 patients with BD. The study found significant effects in improving depression (Cohen’s f = 0.56, *p*  <  0.001), suicidality (Cohen’s f = 0.56, *p*  <  0.001), and anxiety symptoms (Cohen’s f = 0.43, *p* < 0.001) post four ketamine infusions across two weeks. Moreover, the study reported response and remission rates of 35% and 20% across depressive measures at the end of study period (7 days after the last infusion), respectively [45]. Lastly, a small open label study by Permoda-Osip et al. simultaneously investigated antidepressant and pro-cognitive effects of ketamine in patients with BD (*n* = 18). Cognition was assessed through the trail making (TMT) and the Stroop color word interference tests. This study reported statistically significant (*p* < 0.001) improvement in the TMT and Stroop tests 3 days after a single infusion of ketamine (0.5 mg/kg) when compared to baseline. Moreover, the study reported 8 responders (i.e., 50% decrease in depressive measures from baseline) after three days [46].

### 3.5. Safety and Tolerability of Ketamine Use in Bipolar Depression

Treatment with IV racemic ketamine was safe and mostly tolerable with transient side effects (i.e., feeling dizzy, cognitive impairment, dissociation, nausea, headache, odd sensations, flatulence, and blurred vision). These side effects lasted from 30 to 60 min and none of these side effects led to treatment discontinuation [38,40,41,42,43,44,45,46].

There was a report of increased suicidality among two participants in an RCT by Grunebaum et al., with one patient leading to a suicide attempt (post-infusion second month) after IV ketamine in one of the RCTs on BD patients with baseline suicidal thoughts [39]. No other studies reported any sudden increase in suicidal ideation.

Two studies reported manic/hypomanic switching with ketamine infusions among patients with BD. Diazgranados et al. reported one patient in the ketamine group and one patient in the control group (IV saline) who developed manic symptoms after infusion in a total of 18 patients enrolled. However, the study did not delineate patient-specific characteristics or time period for the occurrence of mania [37]. Moreover, the real-world study by Fancy et al. described three patients (out of 66) with BD who developed hypomania after the third or fourth ketamine infusion. All these patients were taking antidepressants alongside IV ketamine treatment [45].

## 4. Discussion

According to our review, IV ketamine demonstrates weak preliminary evidence of efficacy, tolerability, and safety in persons treated for bipolar depression. Moreover, ketamine infusions did not appear to result in a higher rate of manic/hypomanic induction, dissociation, or psychosis when compared to persons with major depressive disorder [26,27,47]. Nevertheless, a limitation of the extant literature is that few studies are controlled and most of them are limited to single infusion studies, while the remainder are largely open-label studies.

The therapeutic management of BD includes primarily three steps of treatment: acute treatment to abate mania/hypomania or depression, maintenance treatment to prevent manic/hypomanic or depressive episodes, and the treatment of subthreshold affective symptoms as well as cognitive impairment [32]. As such, there is some evidence that IV ketamine is effective in the treatment of the acute phase of bipolar depression. However, there is little to no evidence of its use in maintenance treatment and optimization of residual symptoms. Moreover, although IN esketamine is FDA-approved for unipolar depression with extensive data around its long-term use, no such studies exist in the purview of bipolar depression. This leads to a dearth of treatment options in patients with bipolar depression who are not responsive to current treatment strategies (i.e., combination of mood stabilizing agents and antipsychotics) [32]. It has been estimated that up to 25% of patients with bipolar depression who do not respond to initial two-treatment interventions are often termed to have treatment-resistant bipolar depression (TRBD).

As of now, there is no FDA-approved treatment for TRBD, leading to significant emotional, somatic, functional, and financial toll among patients [17]. Furthermore, the lack of consensus definition of TRBD further complicates the spectrum for treatment of patients resistant to polypharmacological combination treatments. As such, the scientific community should acknowledge and further define the TRBD domain that will eventually lead to better evidence synthesis for treatments such as ketamine and psilocybin [17]. Lastly, all the current interventions for bipolar depression take time to ameliorate depressive symptoms and this lag in response leads to only a fraction of patients reporting any improvement in the first week of treatment [17,32]. There is a dire need to study therapeutic targets that may work rapidly with effects in a short period of time [18]. 

Our review suggests that IV ketamine can help ameliorate depressive and suicidal symptoms in a shorter period of time (anywhere from one day to a week) among patients with apparent TRBD. However, there was a report of an increase in suicidal ideation in two participants in an RCT by Grunebaum et al. after ketamine infusion [39]. Although it appears to be an isolated event, future research should focus on tolerability and long-term use of IV ketamine in patients with BD presenting with suicidality. Moreover, there is a need to differentiate the anti-suicidal role of ketamine across BD-I, BD-II, and cyclothymic patients. As of now, preliminary evidence suggests the cautious use of IV ketamine in BD patients to reduce suicidal ideation with constant post-treatment monitoring may be considered in select cases.

Moreover, there is some weak evidence from a real-world study that IV ketamine might be beneficial in improving cognitive deficits among patients with BD [46]. Overall, IV ketamine was mostly tolerable in the majority of patients within our included studies. However, there is an important caveat when it comes to manic/hypomanic switching. It appears from current literature that IV ketamine might lead to manic/hypomanic switching in patients who are currently on antidepressant treatment. Three out of five RCTs tapered the BD patients of antidepressant treatment before infusing ketamine, and hence the use of IV ketamine and antidepressants together remains poorly understood. Even though the evidence is lacking, it is clinically suggested that patients should be put on mood-stabilizing agents (e.g., lamotrigine, valproate, lithium, etc.) and tapered off antidepressants before initiating treatment with ketamine infusions. In a case where antidepressant plus ketamine treatment is needed, extra monitoring might help avoid any adverse event.

### 4.1. Future Research 

There is a relative lack of adequately powered RCTs in bipolar disorder evaluating the role of IV ketamine or IN esketamine in treating psychopathological domains of bipolar depression. Future researchers should aim to discern the therapeutic usefulness of glutamatergic agents in the acute phase of bipolar depression and maintenance treatment of bipolar disorders. Future studies should include a large, complex sample, as well as the use of validated self-report measures to fully understand the role of ketamine in preventing future depressive episodes. It is important to map out clinical predictors that can determine greater effectiveness and/or risk of manic/hypomanic switching among patients with BD. Moreover, although ketamine treatment appears to be safe in BD patients, its safety and tolerability regarding manic/hypomanic switch should be further explored. Lastly, it may also be necessary to consider the potential confounding factors that can arise when studying a patient population with multiple comorbidities, and on many types of psychotropic medications. This could be a limitation to be vigilant of. These future studies are essential to understand the mechanisms of glutamatergic agents in bipolar depression and TRBD.

Although we excluded the studies that had both unipolar and bipolar depression patients, there are some real-world studies including a mix of mood disorder (both unipolar and bipolar depression) patients providing us with some prefatory evidence related to effectiveness of IV ketamine in treating select domains of bipolar depression. These studies have shown some benefits in treating anhedonia, a mood disorder with mixed features, anxiety, and functional deficits (i.e., work productivity and attendance) often being shown by patients with BD [48,49,50,51,52]. These studies can act as a starting point for future researchers to design robust clinical trials to better understand whether ketamine infusions can lead to betterment in patient reported outcomes (e.g., sleep, functional outcomes, emotional disturbances, etc.).

### 4.2. Limitations

Our scoping review is limited primarily due to moderately low quality RCTs and real-world data. The overall quality of evidence poses serious limitations as most of the studies included in the synthesis had a small sample size and a short follow-up period that could study only acute effects. The lack of blinding in the open label studies failed to rule out placebo effect. The varying gender ratio and mixed sample populations (including hospitalized patients, patients with TRBD, and self-sponsoring community clinic patients) further limits the generalizability of results. The missing outcome data and lacking statistical calculations for outcomes in RCTs further diminished our ability to synthesize the exact antidepressant efficacy of IV ketamine use in BD patients. Moreover, there was no post-trial assessment for the success of blinding among control groups in the included RCTs. The blinding techniques varied between IV saline and IV midazolam that could have further reduced the validity of these studies with results that might not be exactly additive for a review. As such, more operative blinding techniques are needed to conduct robust RCTs with ketamine/esketamine treatments in patients with BD. Therefore, findings across efficacy/effectiveness and safety profiles of ketamine from our review should be interpreted cautiously when it comes to the use of ketamine in patients with BD.

## 5. Conclusions

Our review suggests that IV ketamine is a promising acute treatment in adults living with bipolar depression. There is also some preliminary evidence that IV ketamine might have significant anti-suicidal effects in select BD patients presenting with suicidality. However, the cited evidence is limited to short-term studies with most of them being pilot projects. The findings, thus, prove the feasibility of related research and the safety of short-term IV ketamine treatment with minimal risk of manic/hypomanic switching. The evidence is not sufficient to draw clinical guidelines comprehensively. The limitations of the studies made it difficult to rule out a placebo effect, and whether the antidepressant response is sustainable or transient remains unknown too. Furthermore, to translate the findings to clinical practice, the selected sample in the future studies needs to be more representative of the general population. The wide exclusion criteria, acuity of depression, concurrent administration of other pharmacotherapies, and limited sample size in the current literature highlights the need for more comprehensive studies to draw important conclusions regarding the effectiveness and feasibility of ketamine use in clinical practice.

Overall, extant evidence suggests the potential of ketamine in the treatment of bipolar depression and select comorbidities (e.g., anxiety, cognitive deficits, substance use disorders, etc.) in bipolar disorder. Larger multi-infusion studies in well-characterized cohorts of persons living with bipolar disorder are needed to further inform treatment decisions. In the interim, the use of IV ketamine in centers with core competencies in safely delivering this treatment may be considered across difficult-to-treat patients with bipolar depression. 

## Figures and Tables

**Table 1:** Characteristics of included studies.

Sr. No.	Lead Author	Study Type	Total Participants	Intervention (Dosage)	Control	Concomitant Medications	Primary Objective	Psychometric Tool Used	Findings	Any Report of Hypomanic/Manic Switching	Limitations
1.	Zhuo et al., 2020	Open label study (3-week)	38 patients with treatment-resistant bipolar disorder (TRBD); 22 males and 16 females.	0.5 mg/kg; Total 10 infusions	None	Patients continued their original medication regimen including mood stabilizers and antipsychotics.	Therapeutic effects and associated brain alterations following multi-infusion ketamine augmentation in patients with TRBD.	17-item Hamilton Depression Scale (HAMD-17)	Significant differences in HAMD scores after 1 week. (mean reduction = 49.8%, *p* < 0.05). However, relapse of symptoms in 2nd week. By the 21st day, more severe depressive symptoms were reported compared to the baseline. (Baseline HAMD score: 36.5 ± 2.8 and at 21st day: 39.0 ± 2.4).	None reported	Use of self-comparison (no control). Short duration (3-weeks). Excluded patients with suicidal ideation.
2.	Permoda-Osip et al., 2015	Non-randomized, uncontrolled trial	18 patients with baseline Hamilton Depression Rating Scale (HDRS) score more than 18 (4 males and 14 females).	A single dose of 0.5 mg/kg over 45 min	None	Mood stabilizers throughout the duration of the study. Antidepressants were stopped 7 days before infusion.	To measure the change in neurocognitive performance before and on the 3rd day after a single dose of ketamine infusion in patients with a diagnosis of bipolar disorder and in depressed state taking mood stabilizing drugs and to correlate it with the antidepressant effect of the intervention.	17-item Hamilton Depression Rating Scale (HDRS) Neurocognitive tests: Trail making test (TMT) and Stroop color-word interference test	The HDRS scores were reduced by an average of 11 points on the 3rd day and by 12 points on the 7th day post-infusion (24 ± 5, 13 ± 6, and 12 ± 7, respectively). Eight patients had at least 50% reduction of HDRS scores on the 7th day compared to the baseline. Performance on neurocognitive tests improved significantly on the 3rd day after infusion. The degree of improvement in the neurocognitive test scores correlated positively with the degree of baseline impairment on the tests.	None reported	Small sample size. No control group. Possible practice effect on cognitive test performance (with no control group to control the bias). Discontinuation of antidepressants 7 days before the intervention might have fluctuated the cognition.
3.	Rybakowski et al., 2017	Open label clinical trial	53 patients (13 males and 40 females) with bipolar disorder with depression score of at least 18 on HDRS Scale.	A single dose of 0.5 mg/kg over 40 min	None	Mood stabilizers throughout the duration of the study. Most of the patients were receiving more than one mood stabilizer. Antidepressants were stopped 7 days before infusion.	To investigate the effectiveness of a single ketamine infusion in patients with bipolar disorder.	17-item Hamilton Depression Rating Scale (HDRS)	13 patients met the criteria for response (50% reduction in HDRS scores) at 24 h and 27 patients met the criteria at day 7. The criteria for remission was met by 8 and 14 patients at day 1 and 7, respectively. The response was significantly more frequent in males than females.	None reported (within the 7-day period)	Open label. Uneven gender proportion. Antidepressants were tapered off only 7 days before infusion.
4.	Grunebaum et al., 2017	Randomized clinical trial	16 patients (10 females and 6 males) with scores ≥ 16 on HDRS-17 and a score of ≥ 4 on the scale for suicidal ideation (SSI).	Ketamine hydrochloride 0.5 mg/kg in 100 mL of saline over 40 min	Midazolam 0.02 mg/kg in 100 mL of saline over 40 min	Patients continued to take the psychometric drugs they were taking except for the benzodiazepines up to 24 h before infusion.	A feasibility study to evaluate the effect of ketamine versus midazolam infusion on suicidal ideation in bipolar depression.	Clinician-rated SSI for suicidal ideation Hamilton Depression Rating Scale (HDRS-17)	There was an estimated decrease of 5.84 points on SSI at day 1 for patients on ketamine compared to the midazolam group (*p* = 0.074). Similar results were reported for improvement in HDRS-17 scale (six-point decrease with *p* = 0.109). 4 out of 7 on ketamine were classified as responders (50% response) compared to 1 out of 9 for midazolam (CI not significant). Similarly, 3/7 in the ketamine group were remitters compared to 1/9 randomized to midazolam.	None reported	Small pilot sample (limited power). Lower than recommended dose of midazolam was used which might have minimized the effect of midazolam.
5.	Zheng et al., 2020	Single arm open label trial	16 patients (13 males and 3 females).	Six intravenous infusions of 0.5 mg/kg ketamine over 40 min on a thrice weekly basis were administered.	Self-control (Results after the first infusion were compared with the results after the sixth infusion).	Patients continued to take the prescribed antidepressant regimen (at least 4 weeks before screening and infusion) along with other psychotropic agents as augmentation.	A pilot study investigating the antidepressant, anti-suicidal effects and safety of six consecutive infusions of ketamine.	Montgomery–Asberg Depression Rating Scale (MADRS)	After 1st infusion: Rate of response and remission reported as 21.1% (95% CI = 0.9–21.2) and 15.8% (95% CI = 0–33.9), respectively. After 6th infusion: Rate of response and remission are 73.7% (95% CI = 51.9–95.5) and 63.2% (95% CI = 39.3–87.0), respectively. Large and significant decreases in both MADRS scores and SSI-part-1 were noted: 5.8, *p* < 0.001 and 0.8, *p* = 0.018. These findings were maintained across the subsequent infusions.	None reported	Small sample size. No control group. Short follow up period of only 2 weeks.
6.	Fancy et al., 2023	Open label Observational Study	66 patients with treatment-resistant bipolar disorder (27 males and 39 females).	Four intravenous infusions of ketamine 0.5–0.75 mg/kg over a 40-min period. Started with 2 doses of 0.5 mg/kg and increased to 0.75 mg/kg in the 3rd and 4th infusion in case of inadequate response.	None	Patients continued to take their prescribed psychotropic medication.	To evaluate the real-word effectiveness of repeated ketamine infusions for TRBD in a community clinic setting.	Quick Inventory for Depression Symptomatology-Self Report-16 (QIDS-SR_16_) Generalized Anxiety Disorder-7 (GAD-7) Sheehan Disability Scale	There was a significant reduction of QIDS-SR_16_ scores from baseline to all subsequent timepoints (*p* < 0.001). In addition, a significant difference was observed in the QIDS-SR_16_ scores from post-infusion 1 to post-infusion 3 (*p* < 0.001) and post-treatment assessment visit (*p* < 0.05). 35% of patients were classified as responders (50% response) and 20% patients achieved remission at follow-up 1 week following the fourth infusion. QIDS suicidality item score decreased significantly over time with treatment (*p* < 0.001). The difference was also significant between post-infusion scores and post-treatment scores (*p* < 0.05). Anxiety scores also decreased significantly from baseline to post-infusion 3 and post-treatment (*p* < 0.05 and *p* < 0.001, respectively).	Treatment emergent hypomania in three patients (4.5%)–might be due to co-administration of antidepressants. No case of mania reported.	No control group. Small sample size. Patients were required to bear the cost of the treatment leading to a potential selection or expectancy bias. Potential confounding by psychotropic drugs or medical comorbidities as study was conducted on patients under treatment at the community clinic.
7.	Diazgranados et al., 2010	Randomized controlled trial	18 patients with TRBD.(12 females and 6 males).	0.5 mg/kg infused in normal saline.	0.9% Normal saline	Patients were only allowed to take lithium or valproate only.	To determine whether an N-methyl-daspartate–receptor antagonist produces rapid antidepressant effects in subjects with bipolar depression.	Montgomery–Åsberg Depression Rating Scale (MADRS)	The effect sizes for change in MADRS were 0.52 (95% confidence interval (CI), 0.28–0.76) at 40 min, 0.67 (95% CI, 0.42– 0.91) at day 1, and 0.22 (95% CI, −0.03 to 0.48) at day 14. The largest effect was seen 2 days after infusion (d = 0.80; 95% CI, 0.55–1.04)	One participant in ketamine and one participant in control group developed mania.	Small sample size. Normal saline could have masked proper blinding. Tapering of some of the current medications might have led to bias.
8.	Zarate et al., 2012	Randomized controlled trial	15 with bipolar disorder (8 females and 7 males).	0.5 mg/kg infused in normal saline.	0.9% Normal saline	Patients were only allowed to take lithium or valproate only)	The efficacy of ketamine infusion in reducing depressive symptoms in bipolar depression	MADRS	Depressive symptoms as well as suicidal ideation significantly improved in subjects receiving ketamine compared to placebo (d = 0.89, 95% C.I. = 0.61–1.16 and 0.98, 95% C.I. = 0.64–1.33, respectively); this improvement remained significant through Day 3.	None reported	Small sample size. Normal saline could have masked proper blinding. Tapering of some of the current medications might have led to bias.
9.	Saligan et al., 2016	Exploratory analysis of randomized crossover-controlled trial.	36 bipolar depression patients (21 females and 15 males).	0.5 mg/kg infused in normal saline.	0.9% Normal saline	Patients were only allowed to take lithium or valproate only.	To study anti-fatigue properties of ketamine in bipolar depression.	NIH-Brief Fatigue Inventory (NIH-BFI)	Ketamine lowered fatigue scores compared to placebo from 40 min post-treatment. The largest anti-fatigue effects between placebo and ketamine was at day 2 (d = 0.58, *p* < 0.05).	One participant in ketamine and one participant in control group developed mania.	Small sample size. Normal saline could have masked proper blinding. Tapering of some of the current medications might have led to bias.
10.	Abbar et al., 2022	Randomized controlled trial	26 participants with bipolar disorder (Gender distribution not reported).	0.5 mg/kg infused in normal saline.	0.9% Normal saline.	Patients continued their as usual medication.	To study the anti-suicidal effects of ketamine in a suicidal crisis.	Scale for Suicide Ideation (SSI)	84.6% (*n* = 22; ketamine) vs. 28.0% (*n* = 7; placebo) reported resolution of suicidal symptoms (SSI < 4). The odds ratio for resolution of suicidal ideation was 14.1 (3.0 to 92.2, *p* < 0.001) in bipolar disorder patients at day 3 post treatment.	None reported	Small sample size. Normal saline could have masked proper blinding. Patients were allowed to take cannabis and other current medications.
